# Developing a framework to evaluate knowledge into action interventions

**DOI:** 10.1186/s12913-018-2930-3

**Published:** 2018-02-21

**Authors:** Sarah Morton, Suzanne Wilson, Sheila Inglis, Karen Ritchie, Ann Wales

**Affiliations:** 1 University of Edinburgh, Edinburgh, UK; 2 Healthcare Improvement Scotland, Edinburgh, UK; 3 SMCI Associates, North Berwick, UK; 4 NHS Education for Scotland, Edinburgh, UK

**Keywords:** Knowledge, Action, Contribution, Evaluation

## Abstract

**Background:**

There are many challenges in delivering and evaluating knowledge for healthcare, but the lack of clear routes from knowledge to practice is a root cause of failures in safety within healthcare. Various types and sources of knowledge are relevant at different levels within the healthcare system. These need to be delivered in a timely way that is useful and actionable for those providing services or developing policies. How knowledge is taken up and used through networks and relationships, and the difficulties in attributing change to knowledge-based interventions, present challenges to understanding how knowledge into action (K2A) work influences healthcare outcomes. This makes it difficult to demonstrate the importance of K2A work, and harness support for its development and resourcing. This paper presents the results from a project commissioned by NHS Education for Scotland (NES) and Healthcare Improvement Scotland (HIS) to create an evaluation framework to help understand the NHS Scotland Knowledge into Action model.

**Methods:**

The team took a developmental approach to creating an evaluation framework that would be useful and practical. This included a literature review to ensure the evaluation was evidence-based; adaptation of contribution analysis for K2A project; action research with K2A project leads to refine the work and develop suitable measures.

**Results:**

Principles for evaluation and an evaluation framework based on contribution analysis were developed and implemented on a trial project. An outcomes chain was developed for the K2A programme and specific projects. This was used to design, collect and collate evidence of the K2A intervention. Data collected routinely by the intervention was supplemented with specific feedback measures from K2A project users.

**Conclusions:**

The evaluation approach allowed for scrutiny of both processes and outcomes and was adaptable to projects on different scales. This framework has proved useful as a planning, reflecting and evaluation tool for K2A, and could be more widely used to evidence the ways in which knowledge to action work helps improve healthcare outcomes.

## Background

This paper presents the results from a project commissioned by NHS Education for Scotland (NES) and Healthcare Improvement Scotland (HIS) to inform the implementation of the NHS Scotland Knowledge into Action model by the creation of an evaluation framework. The authors of this paper include the research team (Morton and Inglis) commissioned to investigate and develop the framework, as well as the practitioners (Wilson) and managers (Ritchie and Wales) within the NHS who developed and utilised it.

NHS Scotland, like many other health services [1], aims to equip and empower organisations, staff, patients and carers with the resources, skills and confidence to seek, access and share knowledge and put it into practice, when and where it is needed. However, the way that different kinds of knowledge might be put into practice is often not a simple process. It includes the interaction of people and ideas in very specific contexts [2]. New knowledge interacts with existing knowledge [3] and within the organisational constraints and enablers of change [4].

In NHS Scotland, a Knowledge to Action (K2A) strategy acknowledges some of that complexity and aims to deliver support for evidence-based approaches that have a direct impact on clinical care at the front line. This includes a national knowledge infrastructure in the form of the Knowledge Network from NES guidelines and evidence summaries from HIS and the library services workforce. Building on a Knowledge into Action Review [5] the strategy identifies ways to mobilise this knowledge infrastructure to support the Healthcare Quality Strategy ambitions of safe, effective, and person- centred care. This approach goes beyond supporting practitioners in accessing and organising information, focussing on also enabling them to apply knowledge to frontline practice to deliver better healthcare, and embed the use of knowledge in healthcare improvement. This project sought to set up a framework to evaluate K2A in order to understand, develop and evaluate this process.

Acknowledging the complexity of getting knowledge into action presents challenges for evaluation, as discussed in a growing body of literature on this issue [6]. These challenges include attributing change in practice to specific knowledge services or interventions; dealing with different timescales of knowledge production versus practice decision-making; and understanding the relevant contextual issues [7].

The challenge of timing K2A impact evaluation rests on addressing the pay-off between the reliability and availability of evidence in the short and longer term [8]. Practitioners are more able to recall their knowledge use processes immediately, but the significance of any use of evidence will emerge into system-wide impacts only over a much longer time-scale, at which point is is very difficult to unpick the role that knowledge played in any emerging changes in practice.

In a complex, interactive model of K2A as described above, where different kinds of knowledge are incorporated with existing beliefs and understandings to inform healthcare decisions, issues of attribution are particularly difficult for evaluation. Can it ever be reasonable to attribute change to specific K2A interventions? The concept of contribution rather than attribution [7] has been used to aid understanding of knowledge into action, suggesting a view of knowledge as one factor amongst many that influences outcomes.

Contextual analysis has also been embedded in the evaluation framework developed here, as other studies have shown that the specific context for any K2A process (like the immediate team involved, the setting, political priorities, current practice, guidelines etc) is fundamental to how knowledge is used. Bell and colleagues [8] suggest that better analysis of context can help illuminate attribution issues. They recommend that approaches should be complexity-informed, focus on networks and relationships, and take account of context.

Evaluation of K2A, like other evaluation requires clarity of purpose, definition of impacts of interest, and clear methods. The purpose of evaluation might be accountability, assessing value for money for the public purse, better understandings of research use and impact, auditing evidence-based policy and practice, or more recently as part of measures to determine funding [7]. In the case presented here, learning and accountability were key drivers of the evaluation approach, with value for money as a secondary interest.

### Context

NES and HIS Strategic Review Getting Knowledge into Action to Improve Healthcare Quality [9] of the NHS Scotland knowledge infrastructure aligned the K2A work with the aims of the Quality Strategy [10] in order to ensure that K2A was supporting improvement in patient outcomes. This was informed by the US Institute of Medicine [11] and the Francis Report [12], both of which highlight the need to manage knowledge to improve outcomes, and reduce harm, waste and inequity.

The Strategic Review [9] recommended the development of K2A work to improve learning, address health and social care integration, continue translation of knowledge into frontline practice, and to continue to develop strategic partnerships.

**Fig. 1:**
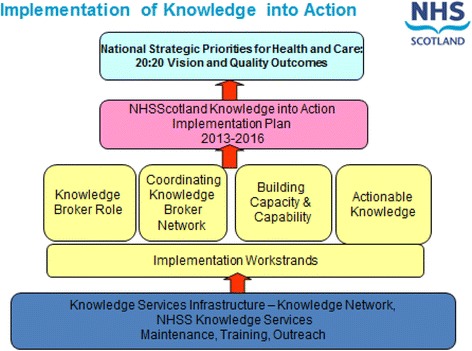
The NHSScotland Knowledge into Action model

In response to this recommendation, NES has developed a sophisticated Knowledge into Action Model (Fig. 1). This provides a vision of a coordinated network of knowledge brokers (people with a role to support knowledge use), integrated with clinical and improvement teams, which aims to help to improve clinical practice and better health outcomes by:

- Delivering evidence search and summary services– combining research with evidence from the experience of teams, patients and carers.
- Delivering knowledge in actionable formats – for example care bundles, decision aids, pathways, decision support.
- Supporting person to person exchange and dissemination of knowledge, through methods such as communities of practice.
- Building organisational capacity and culture for use of knowledge through leadership, and building knowledge management roles and skills.

The NES/HIS Knowledge into Action Strategy has a broad definition of knowledge that includes: quality assured, published research; ‘grey’ literature; knowledge from different kinds of learning, including practice based small group learning and short modules; and knowledge from practice, experience and discussion. The focus of the strategy is on enabling practitioners to combine different kinds of knowledge to inform care.

### Research questions

This paper sets out how an evaluation approach was developed that would be a good fit with this approach to K2A, be pragmatic and based on the best available evidence. The project was commissioned specifically to design of an evaluation framework that would allow testing and evaluation of K2A to enable learning about effectiveness.

The overall study sought to understand the role of different kinds of evidence in healthcare decision-making, whilst the focus of this paper is on the narrower question of establishing an evaluation framework. Specifically:

*What future testing and evaluation might be achieved with support from NHS Education for Scotland and Healthcare Improvement Scotland to fully consolidate successful approaches to combining knowledge in NHS Scotland?*

The rest of this paper reports on the methods to develop an evaluation framework, and discussed how this was applied to a practice example.

## Methods

This project took a developmental approach to creating an evaluation framework that would be useful and practical, whilst also acknowledging complexity and addressing the issues outlined above [13]. This approach was informed by the specific commission from NES and HIS that the evaluation framework should be designed to:

- Support outcomes-focused planning and measurement at all levels of K2A implementation:
  - ○ Development of national infrastructure for Knowledge into Action
  - ○ Local implementation by individual Boards
  - ○ National deployment of Knowledge into Action in three to four projects to support national healthcare priorities
- Develop the outcomes chain measurement model previously used in some smaller K2A projects that had tested new ideas.

The previous outcomes chain measurement model was an adaptation of Contribution analysis [14]. This was a good fit with the outcomes-focused approach of the NHS K2A strategy. Contribution analysis seeks to identify the contribution of an activity or programme to high-level outcomes [15]. It does this through 6 steps: 1. Analysing context; 2 develop a logic model for the programme, 3- assess assumptions and risks, 4- identify evidence and gaps in evidence, 5- collect more evidence 6- write a contribution story or outcome-focussed report.

The method was an action-orientated approach to designing a mode of evaluation for K2A. It involved:

- A thematic literature review to identify key issues and promising approaches in evaluating K2A
- Integration of findings into an evaluation framework in consultation with relevant K2A staff and managers
- Further refinement and testing of the approach carried out through the use of the example presented in this paper.

### Literature review

A literature review investigated the questions: What are the key issues in evaluating K2A initiatives? What approaches to K2A evaluation have been taken?

The approach to the literature review was pragmatic and thematic, building on current literature along with findings from a systematic review on evaluating K2A [16]. This allowed for the identification of key issues and themes as a starting point for approaching evaluation. The focus was on empirical work relevant to the NHS Scotland K2A model, with an emphasis on frontline practice and evaluation. A peer review process was used to ensure good relevance and coverage, which identified eHealth and educational approaches relating to K2A as relevant. This involved a team of four peers, with academic and practical expertise, who reviewed the search strategy, the draft literature review and the final evaluation approach.

Reviews in this area are challenging as search terms such as ‘knowledge’, ‘action’, ‘evaluation’ and ‘impact’ return such huge numbers of citations. In addition, this is an interdisciplinary area, and traditional disciplinary boundaries do not always help narrow the search. The Google Scholar and BioMed databases were searched for relevant articles by using key words. The focus was on terms including “knowledge into action”, “ehealth”; “knowledge into action broker”; “knowledge into action model”; “evaluation knowledge to action” and “contribution analysis clinical practice”, and focusing on publications after 2011 (the previous review carried out by NES/HIS) and 2012 (date of research).

Criteria for inclusion in the literature review were initially on relevance to the research questions, with quality criteria applied after identification. Identification of relevance was informed by the parallel and iterative consultation process and undertaken at the point of review [17]. Identified literature was scanned, and publications which appeared to use empirical research to explore or test a K2A model were accessed and examined. The references in relevant literature were then followed up as a way of tightening the boundaries of the review, until a point of saturation [18] was reached. 163 relevant publications were identified.

A ‘snowball technique’ was used to search for relevant grey literature through Google. Because the existing literature is so vast and diverse (and often not relevant to our research questions), the search was limited to 2011 to date (following the previous review to 2011) and to the first 10 pages of results. The literature review was supplemented with documentary analysis of relevant policy papers on NHS Scotland approach to K2A, improvement and quality assurance, and Scottish Government documents on knowledge for healthcare.

### Development of the evaluation framework

Themes from the literature review, discussion with peer reviewers, and consultation with HIS and NES staff involved in the development and delivery of K2A projects led to the development of two specific products: 1) articulation of a set of evaluation principles for K2A evaluation, and 2) a framework for evaluation of K2A initiatives.

Evaluation principles were drawn from key lessons from the literature, and were developed and reviewed by the peer review group. These guided the evaluation process and helped to shape the kind of approach that would be effective for evaluation of K2A.

In order to develop the evaluation framework, the research team worked with NES and HIS to establish the framework for two key projects as a proof of principle, and to help start to design suitable indicators. One of these projects has been used as an example in this paper.

The literature review highlighted key components of successful K2A evaluation that were included in the framework and evaluation in a number of ways:

1. That there should be a theory-based approach to the evaluation
2. That case studies would be the most appropriate focus of study
3. That mixed methods would be needed to evaluate K2A
4. That it would be important to use methods suitable for the level of complexity of the work

These are elaborated below including details of how they were incorporated in the K2A framework.

There is general agreement that case studies will often provide the best approach to evaluation of knowledge into action. In order to develop case studies a workshop was held with lead officers for a number of K2A projects. During this workshop a specific kind of theory of change [19] (referred to as an ‘outcomes chain’) was articulated for each K2A project using a Contribution Analysis framework adapted by Morton [2]. This framework was suitable as it built on the literature, was pragmatic and also utilised the outcomes-focussed approach that built on previous work. Following the workshop, HIS lead officer refined these frameworks for each project. The researchers and HIS staff worked together to develop indicators for the projects relevant to the different levels in the evaluation framework. These were reviewed as data was collected and discussed with a wider group of stakeholders to validate the measures and overall approach.

The literature had indicated that interviews are often the most useful source of information for K2A evaluation [20], however, mixed methods have proved particularly useful in dealing with different timescales, and as a way of identifying knowledge users for further follow-up [16].

Promising approaches and tools for evaluation of K2A included pre and post intervention survey questionnaires, focusing on clinician knowledge or reported behaviour [21–25]. More detailed approaches involve surveying patients, or extracting patient data from electronic records [26–29]. A few studies have used focus groups to collect qualitative data from clinicians regarding their views on the effectiveness of an intervention. Many of these (especially those relying on surveys of clinicians’ knowledge only) may not be appropriate for more complex interventions and would not provide information on how the K2A activities have or have not had an impact on longer term outcomes e.g. improvements in the quality of care from the perspective of services users or patients.

A pragmatic approach was used in the model developed here, incorporating routinely collected data with feedback and evaluation. This includes some quantitative measures (descriptive), and some qualitative feedback. The evaluation framework created categories for data collection and combining. One example is used in this paper to illustrate the results of this process.

The project passed a level one Ethics assessment within the University of Edinburgh [30] as: “the study does not present any complex ethical issues and does not require further scrutiny.”

## Results

Drawing together the literature, and in consultation with an expert advisory group, the following evaluation principles were established.

**Box 1:** Evaluation Principles for K2A

The K2A Evaluation Process should:
• **Include ‘criteria for success’ from different levels of the system** (micro/meso/macro) and include the views of all relevant actors (patients, practitioners, managers, policy-makers), and link these to the problem definition phase of the K2A process • **Be easy to use** and enhance the planning and implementation process rather than detract from it • **Link K2A activities to wider outcomes**, **whilst also seeking to understand processes, relationship and capacity building**, and be able to provide some evidence of the contribution made by K2A to these • **Have a clear approach to K2A based on understanding of the knowledge utilization processes** • **Acknowledge that there are many influences on healthcare outcomes**, to which K2A provides a contribution. • **Provide evidence about the effectiveness** of different K2A processes to enhance learning about K2A and contribute to the literature

**Fig. 2:**
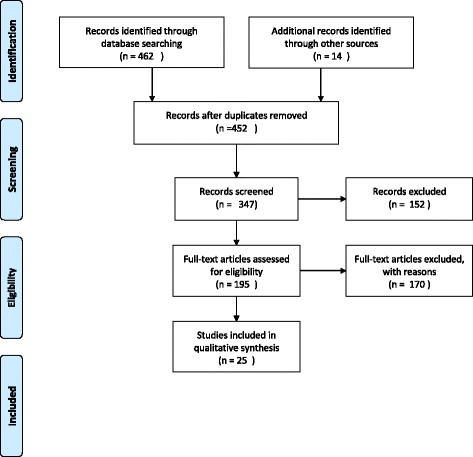
Flow diagram for thematic literature review

In order to operationalise these principles, and building on learning from the literature, an approach using an adapted version of contribution analysis [31] was developed for the evaluation of K2A. Contribution analysis is one example of theory based evaluation [32]. This approach requires articulation of the intentions of a programme by those involved in delivering and planning it, through the setting out of an ‘outcomes chain’. This helps address the issues of linking processes and outcomes, and is thus suitable for complex systems [33]. The evaluation recognised that only the activities, inputs and outputs will be under the direct control of the programme. Figure 2 is commonly used by NES and HIS to explain one of the issues with their work by setting out spheres of influence. These help explain how any programme will have some influence on those individuals and communities directly contacted but will only have indirect influence on the wider communities who might be expected to use or benefit from the programme. In addition, medium and longer-term outcomes will be impacted on by external factors such as the readiness of the context, pre-existing practice and beliefs or the social and political environment. Therefore, the impact of any given activity or output on outcomes has to be considered in the context of these spheres of influence.

In terms of the evaluation of K2A, a contribution analysis approach allowed for:

- A context specific problem-focused approach to planning and evaluation
- A clear method of linking K2A activities to wider outcomes
- The potential to aggregate from project-level data to evaluate programmes
- A pragmatic approach which can help with planning, encourage reflexivity and create learning communities which will enhance planned K2A processes

Figure 2 below shows the K2A evaluation framework that was developed using this approach. It sets out a series of processes through which K2A activities can be linked to outcomes. It’s development and application is discussed below.

### Outcomes framework development

The first step in this evaluation approach is to set out an outcomes chain for the specific K2A work being evaluated. Assessing risks and assumptions for each step in the results chain is then carried out to allow for assessment and inclusion of external factors that may influence any K2A approach. It can also help to identify suitable indicators. For example, if the K2A project assumes good reach of key stakeholders, then the extent this is achieved must become an indicator.

Whilst an outcomes chains may appear linear, it is important to include risks and assumptions across the system to address complexity, and acknowledge that events may not occur in a linear way (as explained elsewhere [2]). It can be helpful to consider the outcomes chain as a navigation tool through complex systems, plotting the links between evidence and action, allowing for change to occur at different times and for feedback loops to be created and included in the model.

**Fig. 3:**
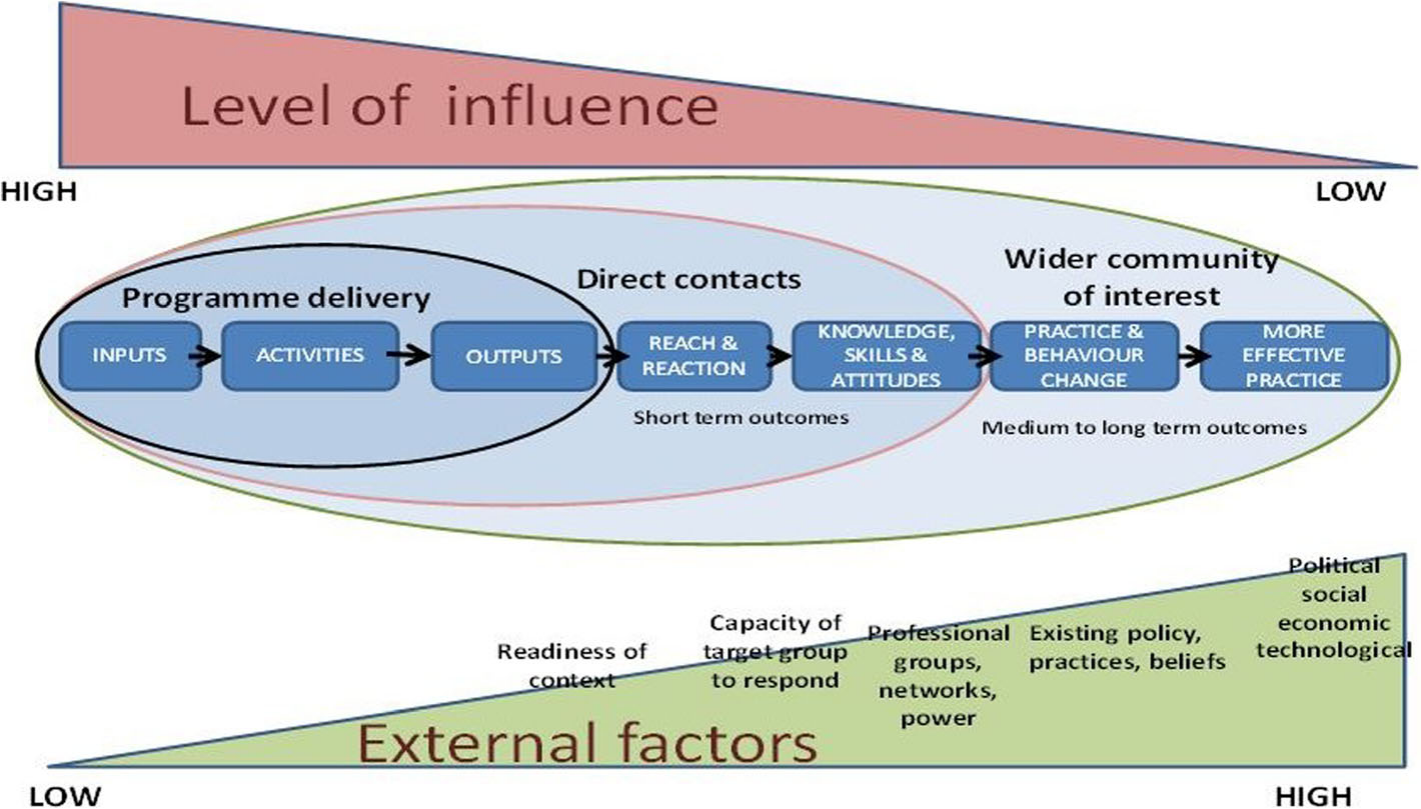
Outcomes Chain for Knowledge into Action (Adapted from Montague by Health Scotland)

The evaluation framework used to assess the K2A programme of work was built at 2 levels – firstly a strategic outcomes framework set out the outcomes chain for K2A work in general, (see Fig. 3). Nested below were outcomes chains for four specific K2A national projects. One of these has been used in this article to illustrate the efficacy of the approach.

Figure 3 shows the strategic outcomes framework and articulates the K2A approach as set out NHS and NES K2A model. It articulates how K2A four national K2A projects are intended to contribute to healthcare outcomes through the processes their reach, how intended audiences react, their changes in knowledge and skills that will underpin any behaviour and practice changes that are needed to contribute to healthcare outcomes.

This strategic level outcomes chain was then developed into specific case-based outcome chains for each of the four national K2A projects using the following process:

1. **Identify K2A activities fit for the context**. In this case key K2A processes identified within the K2A model as appropriate (search and synthesis, actionable knowledge, relational knowledge use, capacity and culture, knowledge brokerage and library resource transformation).
2. **Identify changes in the behaviour, practices and outcomes that K2A process aims to achieve**. What is the problem the approach seeks to address, and how will the activities tackle it?
3. **Link each step from activities to outputs using the categories in the model.** These focus on the process of uptake, learning and behaviour change. Particularly important is consideration of who is engaged, including analysis of gaps in engagement, acknowledging that uptake is the cornerstone of any further knowledge use or impact.
4. **Assess risks and assumptions.** Here some of the existing knowledge on what helps and hinders K2A can be included to inform the analysis. For example, is the approach timely and relevant to user’s needs, does it fit or challenge their current thinking? Validate risks and assumptions through ‘critical friends’ – others who know the setting well and can comment on the robustness of this analysis.
5. **Devise indicators for the outcomes chain.** An indicator set has been developed for K2A projects that can be drawn on. Set up timescales for data collection and reporting suited to the individual intervention, and taking account of the need to balance respondents ability to recall the intervention with allowing time for change to occur.
6. **Review the process as it develops.** Assess evidence on risks and assumptions, identify weaker areas and develop further indicators, review again, seek external input and utilize ‘critical friends’.
7. **Create a contribution report as required.** Report on the development of the approach over required periods of time.

In order to develop the outcomes chains for each of the K2A projects, the following questions were used:

### Risk and assumptions – Links to indicators

Having developed an outcomes chain, it was assessed for risks and assumptions. This allowed for consideration of the context in which K2A activities were to take place, and helped to identify suitable indicators for the evaluation. For example, research evidence needs to be useful and relevant, and fit with users’ needs or it will not be taken up. Contextual factors, competing priorities or wrong timing are all common barriers for evidence to action [34].

### An example K2A evaluation project

The remainder of this article demonstrates the application of this evaluation approach through the example of one of the K2A projects: Clinical Enquiry and Response Service (CLEAR) which was used to test the framework and explore how it could be used, what measures might be appropriate, and the feasibility of working in this way. CLEAR is a search and summary service provided by HIS. CLEAR aims to provide clinicians with summarised evidence relating to **aetiology, diagnosis, prognosis** and **treatment** queries about patient care. CLEAR is delivered by an information team working to a specific service criteria and a defined method [35]. Table 1 shows an outcomes chain developed for the CLEAR service including data gathered during 2015–16.

**Table 1:** Evaluation framework for NHS Scotland: Clinical Enquiry and Response Service (CLEAR)

	Outcome level	Outcomes chain for CLEAR	Measures for CLEAR	Evidence gathered for CLEAR for 2015–16
*Activity/organisation measures*	Resource *What resources will support the activities?*	Existing team of information professionals and health service researchers. Website developers.	Adequate staffing to deliver service in line with demand. Website availability and accessibility.	Staffing data: adequate staffing to cover demand. No issues reported with website.
	Activities *What K2A activities will take place?*	Training of staff in writing summaries; development of efficient processes; website development	Adequate trained staff to deliver service in line with demand. Website usage.	No training required/delivered during this period.
	Outputs *What will the products/methods/services be?*	Web based evidence summaries answering clinical questions.	Number of requests & responses.	For 2015: 92 enquiries received, 63 responses, (remainder referred or declined).
*Immediate outcomes*	Reach *Which targeted groups are being engaged at micro, meso and macro levels?*	Target audience general practice and staff in remote locations. All NHS Scotland practitioners. NHS Scotland Knowledge Services.	Number of enquiries per board/professional groups.	General Practice: 21 enqs Nursing/Midwid: 12 enqs Hospital Medicine: 11 enqs Allied Health Prof: 10 enqs
	Reactions *How do the target groups react to the service? Satisfaction/timely/relevance/ efficiency…*	Useful and relevant service, which saves practitioners time.	Number of visits to website. Number of repeat users. Users indicate they would recommend the service. Users indicate satisfaction with response received. Users indicate it saves time. Knowledge services staff promote CLEAR.	Google Analytics for 2015–16: 31,311 unique page views; 12,865 unique visits. 18% returning visitors.. 16 survey responses during 2015–16: 100% would recommend service and use again; 100% report query being fully or partially resolved; 100% report their practice being partially or fully informed by the response. 6 reported saving 4–6 h of their time, others gave ad hoc comments to this effect.
*Intermediate outcomes*	Knowledge, Attitudes, skills aspirations *What knowledge, skills, attitudes change as a result of using the activity/outputs?*	Improved practitioner understanding of diagnosis, aetiology, prognosis and appropriate therapies relating to patient care. Increased practitioner awareness of evidence resources available. Increase in skills base within knowledge services.	Users indicate they were presented with new information or are aware of new resources. Knowledge service staff indicate they have improved or acquired new skills.	44% of survey respondents reported receiving new information.
	Practice, behaviour change *What practices/behavious do you expect to change as a result of the activity/outputs?*	Clinical decisions informed by best available evidence. Greater engagement of clinical staff with evidence resources.	Increase in usage of evidence based resources via Knowledge Network. Staff indicate their practice was informed by the information provided.	Athens access statistics. User survey. Annual evaluation report by project manager
*Final outcomes*	More effective practice and wider outcomes *How will practice be more effective as a result of the activity/outputs? What outcomes will this contribute to?*	Improved patient care leading to better patient outcomes.	Case studies of improved care.	Individual follow up with practitioners via survey responses if appropriate.

The table shows in column 2 the questions for developing the outcomes framework that could be used for any K2A project (as detailed previously in Table 2). Column 3 shows how this chain was specified for the CLEAR project- this is an idealised version of how the project would lead to outcomes. Column 4 shows the measures that were agreed to test each level in the outcomes chain. Column 5 show the evidence gathered against these measures during 2015–16.

**Table 2:** Outcomes chain development

*Activity/organisation measures*	Resource	*What resources will support the activities?*
	Activities	*What K2A activities will take place?*
	Outputs	*What will the products/methods/ services be?*
*Immediate Outcomes*	Reach	*Which targeted groups are been engaged at micro, meso and macro levels?*
	Reactions	*How do the target groups react to the service? Satisfaction/timely/ relevance/efficiency…*
*Intermediate outcomes*	Knowledge, Attitudes, skills aspirations	*What knowledge, skills, attitudes change as a result of using the activity/outputs?*
	Practice, behaviour change	*What practices/behaviours do you expect to change as a result of the activity/outputs?*
*Final Outcomes*	More effective practice and wider outcomes	*How will practice be more effective as a result of the activity/outputs? What outcomes will this contribute to?*

Risks and assumptions were discussed in order to underpin the project logic, and to underpin the idealised version of the outcomes chains. In the CLEAR test case given in this paper, assumptions were made that practitioners would value evidence based approaches to their practice and would access the enquiry service when they were uncertain about a clinical decision or recognised a gap in their knowledge, and that they would believe it would save them time. The biggest risk identified for the enquiry service was the potential to report inaccurate information. By recognising this, mitigation could be put in place in the form of staff training and quality assurance processes.

Utilising these assumptions and risks helped to suggest criteria for monitoring and evaluation, for example, asking for feedback about the relative value of the evidence provided by the service,

Potential indicators for K2A, were brought together in recent paper by Mansfield and Grunewald (2013) [36]. This approach to providing indicators across different K2A functions has provided the basis for the indicator suite developed for the K2A evaluation approach for NHS Scotland (Table 3).

**Table 3:** Foundation for K2A indicators (adapted from Mansfield & Grunewald, 2013 [36])

Type of activity	Indicator	Outcome level^a^
Online community of practice		
	# of members and types against target	3
	# of contributions (differentiated by content type, such as discussion, file, blog etc)	3
	# of views of different content types	3
	Distribution of member participation (contributors, views etc)	3
	Would target audience miss if discontinued?	3/4
	# of conversations you have had as result of the community	5
	Have you talked to someone you did not talk to before?	5
	Have you worked with anyone outside the portal that you met here?	5
	Can you give an example of what the CoP enabled you to do?	Potential for 3–6
Knowledge services		
	# of requests for information by target audiences	3
	% of repeat requests from particular stakeholders/service users	4
	Would you recommend the service to others?	4
	% feedback from users	3/4
	Knowledge provided is of good quality and meets my requirements	5
Knowledge products		
	# knowledge product created	2
	% users who rate knowledge products as excellent/useful	3
	# citations of knowledge products	5
	# people having read/used knowledge product	3
	# recommendation of knowledge product	4
	Usefulness of knowledge product (likert item 1–5) as perceived by target audience	5
	Use in practice as reported by target audience	6
Knowledge sharing/brokering		
	I feel encouraged to share knowledge with my colleagues	5
	I have shared knowledge with a colleague at least once a week	5
	I know precisely who in my organisation has the specific capacity to help me identify relevant knowledge for my work	5
	I am able to find the knowledge I need quickly and easily	5
	We have structures for team and project work that encourage people to bring forward experiences and insights from other settings	5
	We encourage multiple perspectives and different points of view to emerge	5
Knowledge activities/success cases		
	#% staff who are able to provide an example of how knowledge activities have contributed to a change in practice	6
	#% staff who are able to provide an example of how knowledge activities contribute to local or national level indicators	7
	#% staff who give an example of where learning has improved a policy or programme	6
	Feedback on what would have happened without the knowledge activity	Potential 3–7

^a^7: End Outcomes, 6: Policy or practice change, 5: Capacity, Knowledge, skill, 4: Awareness, Reaction, 3: Engagement, Participation, 2: Activities and Outputs, 1: Inputs

The evaluation generated data of steady monthly uptake of the service (see Table 1 above), and a large number of additional views of each evidence summary highlighting the service was reaching a large audience. The annual report for 2015–16 highlighted that 100% of 16 respondents to the survey would use the service again and would recommend it to a colleague.

It is more challenging to monitor intermediate outcomes in terms of change in knowledge, attitudes and skills or policy and practice. An on-line survey is sent to all enquirers with the response to their query. During the year 2015–16, 92 enquiries were submitted to the CLEAR service, 63 of which were responded to by the team (others were referred to other services or declined as they did not fit the criteria). Of the 63 enquirers who received an answer to their query, 16 responded to the survey and have provided some good insight into the effectiveness of the work.

Through the data gathered via the survey, 44% of practitioners (*N* = 7) reported they had received new information and 94% (*N* = 15) changed a decision based on information provided to them by the CLEAR service. One survey respondent reported reassuring a patient no further treatment was required based on the evidence received, and therefore preventing over treatment. All other respondents (*N* = 16) reported using CLEAR fully or partially informed their practice and fully or partially resolved their query. Some (*N* = 6) practitioners reported they may have spent between two and four hours finding information to help answer their query, so the service has made considerable time savings for practitioners, which is also demonstrated through comments received via the survey during 2015–16:

“Having this service saved me a lot of time in a service where I am the only one in my profession and don't have a lot of time to conduct literature searches like this which are really helpful in informing treatment.” (Survey respondent)

“I have already recommended this service. It's brilliant and has saved me heaps of time. Thank you!” (survey respondent)

“This service was very helpful because it saved me a lot of time - something which is in very short supply for many practitioners in the NHS” (survey respondent)

Using this approach to evaluating impact of the enquiry service enabled monitoring of indicators in order to react to changes. For example, monitoring the number of enquiries from each NHS board helps to understand how far the targeted audience has been reached and if specific activity, such as marketing, is required in specific Boards. Responses to the user survey indicate whether the evidence is still contributing to changes in knowledge and/or practice and if improvements to processes are required.

As is evident from Table 1, and unsurprising given the nature of immediate and longer-term outcomes, it is easier to evidence outcomes at the activities and engagement end of the outcomes chain where there is a greater degree of control and influence. As the chain moves toward practice changes and final outcomes, many indicators rely on reporting from staff on their observations of the application of K2A tools and the subsequent actions associated with this. The risks and assumptions analysis can help with validity here: if the logic is sound, risks and assumptions have been identified and data collected to show that these have been managed well, then the framework can be judged on the achievement of successful activities and engagement, coupled with some feedback about subsequent change.

Responses to the survey have been low, although those that have responded usually are able to offer rich insights into how the service has helped them develop their practice (in line with findings elsewhere in this field [25]).

Table 3 sets out a indicator suite developed to sit alongside the evaluation framework. This details potential indicators for K2A activities, and illustrates their potential use at different levels of the outcomes chain.

## Discussion: Addressing complexity and K2A evaluation challenges

The framework and approach presented here go some way towards addressing the main challenges of evaluating K2A work in complex systems. It is well suited to this use for a variety of reasons. One of the distinct features of this approach, and important from a complex systems and K2A perspective, is acknowledgment of the key role of networks and relationships (Best and Holmes 2010). This is included in the model in two ways in relation to the different levels of outcomes set out in the outcomes chain (Table 2). Firstly by including an outcome level that focusses on participants’ reaction to any K2A intervention, rather than assuming that engagement will lead directly to behaviour change. If new knowledge does not chime with current issues and practices, it is unlikely to be used, so monitoring at this stage is essential. Secondly, the outcomes chain considers the knowledge and skills that underpin any behaviour or practice change (see Table 1) within the model. For example, it may be possible to find enthusiastic participants who are keen to take up and use the K2A approach, however, without organisational support to develop the required knowledge and skills then further utilisation and potential impact will be limited. This can help to effectively target initiatives to the appropriate level in the system.

Another distinctive and important aspect of using this approach is the process of identifying risks and assumptions along the results chain. It allows for an assessment of the robustness of the chain which will feed into the evaluation process. It immediately creates categories for data collection: for example, in terms of engagement and involvement, an assessment of the potential participants who were or were not engaged and any gaps in participation: essential building blocks of K2A projects. Identifying risks and assumptions sets up monitoring criteria that can be used to ensure impact. For example if enthusiastic participants have no capacity to influence the system, further activities to engage their supervisors, managers or others who have more influence can be devised. Analysing risks and assumptions can help frame suitable questions for any follow-up activity by focussing beyond the expected change set out in the outcomes chain. For example, when analysing change, questions about key factors can be explored with participants, such as: ‘fit’ with current thinking and policy, the extent to which they value the K2A process, and the influence of contextual factors.

The process of planning, evaluating and acting makes this evaluation approach more dynamic and able to accommodate some of the complexity of interactions allowing for the creation of feedback loops within the system, creating a more likely chance of successful outcomes. Similar adjustments can be made if external factors change or have unanticipated consequences, for example: a change in policy direction or a crisis resulting in a change of priorities. This approach creates a cycle of planning, evaluating and learning, helping to acknowledge the complexity of the research-use process and work with it as suggested by Rodgers (2008) [32]. It simultaneously allows for the clear linkage of activities to wider outcomes.

The framework can be used over various timescales, capturing immediate impact, but also medium term and longer-term impact. Data can be assembled along the model at various different stages and as time passes. At the activity and reaction levels, data from project monitoring and feedback can be collected, collated and reported on at regular intervals. Feedback on intermediate and longer term outcomes can be sought at 6-monthly or longer intervals. The framework can be used to keep revisiting whether or not changes can be observed over a suitable timeframe for the specific context of any K2A project. It can be adapted as changes occur in the system (e.g. new policy initiatives or practice priorities), and these can be incorporated.

### Attribution and the counterfactual

Mayne [15] suggests that once an outcomes chain has been set out, data assembled and a ‘contribution (or performance) story’ written, there should be an assessment of alternative explanations of change to address the attribution issue. Bell and colleagues^7^ suggest one way to address this is through respondents’ reflections on what would have happened without the new knowledge/intervention – in this case K2A activities.

However, Patton [13] argues that complexity sensitive evaluation approaches make ideas about the counterfactual meaningless because there are far too many variables in a complex system, and the nature of dynamic interactions emerging into various patterns of activity means that it is difficult to conceptualise counterfactuals in a useful way. If K2A is one element of a number of processes which lead to specific outcomes, but only within contexts where contextual drivers make it useful and relevant, then the idea of being able to assess what would have happened without K2A becomes less meaningful and more speculative.

Mayne’s approach is to utilise outcomes chains to create a reasonable claim about the influence or contribution of the programme, with the robustness of the evidence supporting the logic of the results chain being used to judge the validity of the claim. This approach to K2A assessment could also usefully illustrate why expected impact had not been achieved through the same approach. If participants found new knowledge challenging, if it was counter to current policy trends, this approach could utilise contextual analysis and feedback from participants to show that lack of impact was not related to the knowledge or activities themselves but to the context for K2A. This approach might also be used to suggest impact over a longer time-frame or to re-align activities to address the contextual factors, e.g. through working to address issues elsewhere in the system or rethinking how to challenge entrenched ways of doing things.

### Limitations of the approach

There are inevitably limitations to any approach to evaluation of complex interventions applied within complex systems. The outcomes chain approach requires that a theory of change is formulated which links activities to short, medium and long term outcomes, and that this theory of change should have an evidence base. Where there is a lack of such evidence a theory of change can be tested during the course of the evaluation process, but the development of such theories in the absence of good quality evidence may be difficult. Development of outcome chains may be relatively straightforward for those who are familiar with such analytical processes, but practitioners such as those within health care settings, or in other areas of public service, may find theory building in the realm of knowledge into action more challenging. The identification of outcome indicators and collection and analysis of evidence to allow assessment of success in achieving these outcomes may be similarly challenging to a practitioner group which is more familiar with provision of services than exploring the value of them.

Furthermore, the simplistic question ‘does x work’ whether appropriate or not still tends to permeate some systems, particularly healthcare. Despite evaluation approaches such as the one described here, knowledge into action activities may not be recognised as having sufficient impact to justify assessment. Application of the approach needs to be further tested in a broader range of knowledge into action initiatives in different settings to assess how feasible this is for routine use and how the findings of such evaluations are responded to by policy makers and practitioners alike.

## Conclusion

This approach is different to other K2A assessment frameworks in a number of ways. It doesn’t categorise types of impact, for example types of benefits to specific sectors, instead emphasising processes, allowing for maximum learning for K2A professionals on effective K2A. It can easily be streamlined into planning and monitoring approaches within organisations making it more practical and more likely to be used in reflection and learning. It uses routinely collected data as a starting point for indicators, adding on other methods only where really needed to support claims, making it efficient and inexpensive as an evaluation approach. It sits clearly within the developmental evaluation approach [13], which is distinctive from other K2A assessment frameworks.

The evaluation framework presented here can be used to address several different purposes of evaluation: as the basis for learning and reflection; reporting for accountability or value for money; as the basis of different types of reports to funders or stakeholders.

## Abbreviations

CLEAR: Clinical Enquiry and Response Service
HIS: Healthcare Improvement Scotland
K2A: knowledge into action
NES: NHS Education for Scotland
NHS: National Health Service
